# Supplementary material for: MetaQTL: a package of new computational methods for the meta-analysis of QTL mapping experiments
Source: BMC Bioinformatics. 2007 Feb 8;8:49. doi: 10.1186/1471-2105-8-49 (PMC1808479; doi:10.1186/1471-2105-8-49)
Supplement: Additional File 7 — MetaQTL Package : jar file and tutorial. This Zip archive contains both the MetaQTL JAR file and the files of the tutorial. [file 1471-2105-8-49-S7.zip › org.inra.metaqtl/doc/org/thalia/bio/package-frame.html]

org.thalia.bio


org.thalia.bio

Interfaces 
  
*IBioAdaptable*
  
*IBioAdapter*
  
*IBioAllele*
  
*IBioCross*
  
*IBioCrossTypes*
  
*IBioEntity*
  
*IBioEntityTypes*
  
*IBioGenome*
  
*IBioIndividual*
  
*IBioIndividualTree*
  
*IBioLGroup*
  
*IBioLocus*
  
*IBioOntology*
  
*IBioOntologyTerm*
  
*IBioPopulation*
  
*IOntologyTermProperties*
